# The Current Attitude Toward Death and Hospice Care Among Medical Students in Mainland China

**DOI:** 10.3390/healthcare13162012

**Published:** 2025-08-15

**Authors:** Luo Gan, Yuxin Wan, Yanwei Su

**Affiliations:** 1 School of Nursing, Tongji Medical College, Huazhong University of Science and Technology, Wuhan 430030, China; u202212846@hust.edu.cn; 2 College of Life Science and Technology, Huazhong University of Science and Technology, Wuhan 430030, China; u202112980@hust.edu.cn

**Keywords:** hospice care, attitude to death, students, medical, medical education

## Abstract

Background: This study stems from the perceived need to update skills and training in the process of educating healthcare professionals in light of the needs of individuals and their families. Objectives: This study aimed to assess the prevailing attitudes toward death and hospice care among medical students in China, providing a foundation for implementing hospice care and death education within these institutions. Methods: We conducted an online survey questionnaire with 568 medical students. Results: The results indicate that the overall attitude toward death was more inclined to accepting death neutrally. Gender, place of origin, educational background, willingness to care for terminally ill patients, experience in caring for terminally ill patients, and more will affect the attitude toward death of medical students. Compared to their rural counterparts, medical students in urban areas are more likely to view death as neutral. Instead of reducing fear, death and hospice education made people more likely to avoid situations. All five dimensions of death attitude exhibit a substantial positive connection with attitudes toward hospice care. In comparison to earlier research, medical students exhibit increasingly favorable attitudes regarding hospice care, and their overall perspective on death remains one of natural approval, suggesting that the integration of death and hospice care teaching is progressing effectively in mainland China. Conclusions: Simultaneously, it was discovered that numerous deficiencies required enhancement, including the need for timely feedback and optimization in hospice care instruction and death education, as well as insufficient attention and educational guidance regarding the individual differences and psychological conditions of medical personnel in the future.

## 1. Background and Purpose

With the continuous improvement in social requirements for quality of life, how to provide comprehensive and continuous support for patients facing severe or life-threatening diseases and their families has become a core challenge for China’s medical system. In this context, the importance of palliative care has become increasingly prominent. According to the definition of the World Health Organization, palliative care is a comprehensive medical service model aimed at improving the quality of life of patients and their families [1]. It can effectively relieve the physical, psychological, social, and spiritual pain of patients through early identification and intervention. Importantly, there is no direct causal relationship between the need for palliative care and aging; it is appropriate for patients of all ages, at any stage of the disease, and can be synchronized with aggressive curative treatment [2]. One of the cornerstones of palliative care practice is excellent communication skills. It is not only information transfer, but also a key bridge to build trust, for shared decision-making, and to provide emotional support. It is a core competence that needs to be strengthened in modern medical education. In addition, palliative care considers the family as an inseparable care unit whose support runs through the whole course of the disease and naturally extends to the stage of grief support after the patient’s death, which is the embodiment of continuous care for the family as a whole [3]. Therefore, to develop a future medical reserve that can meet the changing needs of patients and families during the disease journey and truly place “people” at the center of care, China’s medical education system must undergo a profound paradigm shift. Therefore, it is necessary to establish an accurate and comprehensive understanding of palliative care in the curriculum and integrate evidence-based communication skills, family-centered care concepts, and grief support education into the whole process of medical students’ training. The purpose of this study is to provide a theoretical basis and directional reference for such education reform by studying current mainland China medical students’ attitudes toward hospice care and death, and ultimately promote the development of China’s medical service in a more humanistic and holistic direction.

One cannot comprehend life unless one experiences death. Many studies in mainland China have revealed that medical students’ attitude toward death is mainly “natural acceptance”, which is significantly related to the health status of individuals and their families, and the experience of contact with death [4]. The understanding of hospice care among medical students is notably deficient, with minimal enthusiasm; fewer than one-fifth are willing to pursue careers in this field. The primary impediments include occupational stress, low income, and ethical uncertainties [5]. Some studies indicate a positive association between attitudes toward dying and hospice care; nevertheless, the significance of this correlation remains undetermined [6]. International research indicates that medical students are influenced by many religions and civilizations, with those holding religious views (such as Islam and Christianity) exhibiting a greater acceptance of mortality [7]. Arab medical students are more likely to support active intervention, while Western medical students pay more attention to patient autonomy [8]. Meanwhile, it is also important to cultivate the spirituality of medical students. By embracing a patient-centered approach, professionals can use their own humanity and spiritual awareness not to provide answers, but to help patients and families discover their own sources of meaning, strength, and peace in the face of illness and death. This integration of the spiritual dimension is the hallmark of truly compassionate, whole-person care, acknowledging that in healing, we must attend to the soul as well as the body. Despite numerous studies conducted by researchers both domestically and internationally regarding medical students’ attitudes toward hospice care and death, significant deficiencies persist, including a disconnect between theory and practice, as well as an absence of systematic evaluation of the effectiveness and long-term impact of death education and hospice care education [9]. The local education is inadequate, and a curriculum for death education and hospice care, which is relevant for medical students in mainland China, has not been established. The instructional material must be intricately aligned with China’s cultural context and practical clinical requirements [10]. The investigation into the correlation between attitudes toward hospice care and attitude towards death is insufficiently comprehensive, necessitating further exploration of a more refined interaction mechanism and intervention strategies [6]. Due to geographical constraints, many previous studies were limited to examining the hospice care and death attitudes of medical students at a single location or institution, resulting in low generalizability. This study aims to explore the attitudes of contemporary medical students in Chinese colleges and universities regarding death and hospice care on as extensive a geographical scale as possible, expand the horizon of research, and conduct further correlational research to understand the development and popularity of hospice care and death attitude tendencies in mainland China. Furthermore, this study aims to provide a reference for colleges and universities to effectively develop and optimize related courses [11], and provide policy recommendations to meet the needs of clinical practice [12]. It also aims to explore the future trend of the development of hospice care in China to address the challenges of the aging society [13].

## 2. Materials and Methods

### 2.1. Data Collection

This study employs a cross-sectional study paradigm to assess medical students’ attitudes toward death and hospice care, and the influence of current medical education. From April 2024 to September 2024, the questionnaire was published online by the method of convenience sampling. The primary data were sourced from medical students across several institutions and colleges in multiple provinces in mainland China. The inclusion criteria consisted of full-time medical students from mainland China who voluntarily participated with informed consent, while the exclusion criteria included non-medical students from mainland China, healthcare professionals engaged in clinical practice, and individuals with incomplete or logically inconsistent questionnaire responses.

### 2.2. Measures

#### 2.2.1. Assessment of General Information

The general information assessment items attached to the Chinese version of the Frommelt Attitudes Toward Care of the Dying Scale Form B (FATCOD-B) by Wang Liping [14] were referred to. They encompassed gender, place of origin, educational background, only-child status (in this research, it is operationally defined as an individual who had no biological siblings, including full- or half-siblings, throughout their childhood developmental period), personal education, and pertinent experience in hospice care.

#### 2.2.2. Assessment of Attitude Toward Hospice Care

We used the Chinese version of the FATCOD-B scale, originally established by Frommelt [15] in 1991, for assessment. The measure comprises 29 items, and each item is rated on a 5-point Likert scale (strongly agree, agree, uncertain, disagree, strongly disagree), of which 16 items are reverse-scoring items. The remaining 13 items were forward-scoring items, and the scoring technique was contrary to reverse scoring. The total score varied from 29 to 145, with higher scores indicating a more favorable attitude toward hospice care. The Cronbach’s α coefficient for the scale was 0.959, and the content validity was 0.920 among senior nursing students in a medical college in Guangdong Province [14]. The test–retest Cronbach’s α coefficient was 0.766. In addition, it is critical to recognize the distinction that the Frommelt Attitude Toward Care of the Dying Scale (FATCOD-B) assesses a broader and more fundamental construct than an “attitude towards hospice care”. FATCOD-B is designed to measure an individual’s disposition and beliefs regarding the act of caring for any person who is dying, a universal component of healthcare that occurs across diverse settings such as hospitals, long-term care facilities, and homes. In comparison, “attitude towards hospice care” generally refers to one’s views on a specific, formalized philosophy and model of end-of-life care delivery. Therefore, in this study, “attitude towards hospice care” merely refers to Chinese medical students’ views on the concept of local hospice care and their behavioral tendency to care for dying patients.

#### 2.2.3. Assessment of Attitude Toward Death

The Chinese version of the Death Attitude Profile Revised (DAP-R) was adapted by Tang Lu [16]. The scale encompasses five dimensions of escape acceptance (5 items), approach acceptance (10 items), neutral acceptance (5 items), fear of death (7 items), and avoidance of death (5 items), for a total of 32 items. All items were positively scored on a 5-point Likert scale. The higher the average score of each dimension is, the more inclined the death attitude is to this dimension, and the dimension with the highest average score can represent the death attitude tendency. The Cronbach’s α coefficient for the scale was 0.875 among nurses in a hospital in Ningxia [16]. The Cronbach’s α coefficient for the scale was 0.892, and its validity was 0.837.

#### 2.2.4. Data Analysis

The online questionnaire survey outlined its objective and requirements at the outset and was completed by medical students. The SPSS 27 statistical program was utilized for data analysis and processing. An independent-sample *t*-test was employed for two data groups, while a one-way ANOVA was utilized for numerous data groups to examine the factors impacting medical students’ attitudes on dying and hospice care. Simultaneously, Pearson correlation analysis was employed to investigate the relationship between medical students’ attitudes toward death and their attitudes toward hospice care, followed by multiple linear regression analysis of the hospice care attitudes of medical students, leading to the final results.

### 2.3. Ethical Considerations

The study was conducted according to the guidelines of the Declaration of Helsinki, approved by the Ethics Committee of Tongji Medical College (S062), and obtained written consent.

## 3. Results

### 3.1. General Information About Medical Students

We collected a total of 668 questionnaires, of which 568 were valid, resulting in an effective recovery rate of 85%. We excluded questionnaires in which all the checked options were the same or regularly checked options. As can be seen in Table 1, there were 376 females (33.8%) and 192 males (66.2%). There were 262 cases reported from urban areas and 306 cases from rural areas, while 366 cases were not only children. The educational background was mainly undergraduate, with a total of 528 cases. Most of the medical students chose medicine of their own will, accounting for 80.5%, and 422 students were willing to take care of terminal patients, accounting for 74.3%. Over fifty percent of medical students had not enrolled in specialized courses on death and dying, although some relevant topics were included in other courses. Only 101 medical students had taken any special courses about dying and death, while 141 medical students had never been exposed to any knowledge about dying and death. The majority of medical students lacked prior experience in patient and family care, and most had not experienced the loss of a loved one.

### 3.2. Medical Students’ Attitudes Toward Hospice Care

The score for the Chinese version of FATCOD-B was 103.98 ± 9.64. The maximum score was attributed to item 1, “Hospice care is a meaningful thing” (4.36 ± 0.77). Other items with elevated scores included item 4, “Caring for family members of patients during and after death” (4.15 ± 0.77); item 12, “Family members should assist in taking care of the daily life of dying patients” (4.18 ± 0.70); item 16, “Family members need emotional support to accept the behavior change of dying patients” (4.11 ± 0.75); item 4, “Caring for family members of dying patients is a meaningful thing” (4.36 ± 0.77); item 17, “When the patient is about to die, family members should accompany him/her more” (4.16 ± 0.75); and item 18, “Family members should care for and help dying patients to better spend the remaining time of life” (4.26 ± 0.71). The minimum score was recorded for item 19, “not allowing end-of-life patients to make decisions about their daily life care” (2.21 ± 0.98). Other items with low scores included item 28, “Medical personnel have no responsibility to educate family members about death and end-of-life care” (2.43 ± 1.09); item 5, “I do not want to care for a dying patient” (2.62 ± 0.96); and item 15, “I will choose not to be with a dying patient when he or she dies” (2.65 ± 0.99).

### 3.3. Medical Students’ Attitude Toward Death

The average scores for each dimension of the death attitude description scale revealed that neutral acceptance had the greatest score (4.04 ± 0.57), while approach acceptance had the lowest score (2.82 ± 0.74), indicating that medical students had a greater tendency toward neutral acceptance of death. The details are shown in Table 2.

### 3.4. The Influence of Medical Students’ General Information on Their Attitude Toward Death and Hospice Care

The results of this study showed that there was a significant difference in the attitude toward hospice care between the only-child and non-only-child medical students. At the same time, the attitude toward hospice care of the only children was more positive than that of non-only-child medical students. There was a statistically significant difference in the attitude toward hospice care between the medical students who were willing to take care of dying patients and those who were not, with the former having a more positive attitude toward hospice care.

The average scores of different dimensions of death attitude were compared between groups. Initially, gender influenced the inclination towards death attitudes across fear of death, avoidance of death, and neutral acceptance. Male students had a higher tendency towards death fear and death avoidance than female students, while female students showed a greater tendency towards neutral acceptance compared to male students. Both male and female students tend to neutrally accept death. People who grew up in urban areas are more likely to neutrally accept death. Whether they are the only child affects their tendency to fear death and their approach to accepting death. Only children are generally more fearful of death and demonstrate approach acceptance of death. The educational background influences death attitudes, with acceptance progressing from junior college to undergraduate, senior high school, and graduate levels or higher. The status of receiving hospice care and death education in the past can influence avoidance and approach acceptance of death. Individuals who have taken related courses are more inclined to escape acceptance of death and approach acceptance of death. The past experience of caring for end-stage patients and their family members affects the neutral acceptance and approach acceptance of death. Individuals with experience in caring for end-stage patients and their family members have a lower neutral acceptance tendency and a higher approach acceptance tendency. The experience of bereavement in the preceding year affected the death attitudes of neutral acceptance, approach acceptance, and escape acceptance. The results indicated that the more bereavement there was, the more difficult it was to accept death neutrally, and the higher the tendency to approach acceptance and escape acceptance was. The five dimensions of death attitude were all affected by current experience, with fear of death, avoidance of death, approach acceptance, and escape acceptance ranked in declining order: I currently have a terminally ill loved one (life expectancy less than 6 months). > I just experienced the death of a loved one. > I do not currently have a terminally ill loved one. The opposite is true for the dimension of neutral acceptance. The details are presented in Table 3, and *p* values of less than 0.1 were considered to indicate statistical significance.

### 3.5. Correlation Analysis Between Medical Students’ Attitude Toward Death and Attitude Toward Hospice Care

As shown in Table 4, the results showed that the fear of death, death avoidance, neutral acceptance, approach acceptance, and escape acceptance were significantly positively correlated with hospice care attitude.

### 3.6. Multiple Linear Regression Analysis of Influencing Factors in Medical Students’ Attitude Toward Hospice Care

Table 3 and Table 4 present factors influencing the attitude toward hospice care of medical students with a single factor *p* < 0.05 as described in the previous section: only child or not (1 = yes, 2 = no, *p* = 0.028), willingness to care for terminally ill patients (1 = yes, 2 = no, *p* < 0.001), death avoidance (*p* = 0.000), fear of death (*p* = 0.000), neutral acceptance (*p* = 0.000), approach acceptance (*p* = 0.000), and escape acceptance (*p* = 0.000) were the independent variables, and the scores of the Chinese version of FATCOD-B were the dependent variables for multiple linear regression analysis in Table 5, with stepwise regression analysis conducted twice (*p* < 0.01). However, as highlighted in the Discussion Section, the factors listed in (1), (2), (3), (4), (5), and (6) influence attitudes toward death, but not hospice care. So, we did not include these factors as explanatory variables at the beginning of our multiple regression analysis. As shown in Table 6, the results indicated that only four variables were included in the final regression equation: fear of death, death avoidance, neutral acceptance, and willingness to care for dying patients. The Durbin–Watson score was approximately 2, indicating that the residuals in the regression model were deemed independent. The adjusted R^2^ value was 0.301, indicating that the proportion of the four variables in the model explaining the influencing factors of hospice care attitude was 30.1%. The overall significance of the ANOVA table was less than 0.01, indicating that the model was valid. The corresponding VIF values were approximately 2, which indicated that there were no collinearity issues. Fear of death, death avoidance, neutral acceptance, and willingness to care for dying patients were positively correlated with the score of hospice care attitude; namely, the higher the fear of death score, the more positive the hospice care attitude; the higher the death avoidance score, the more positive the hospice care attitude; and the higher the neutral acceptance score, the more positive the hospice care attitude. Medical students who are willing to care for dying patients have a more positive attitude toward hospice care. According to the standardized coefficient Beta, the degree of influence on the hospice care attitude score was in the order neutral acceptance > fear of death > death avoidance > willingness to care for dying patients.

## 4. Discussion

Dying patients face the threat of death all the time. The importance of hospice care is clear in improving the quality of life for dying patients and allowing them to spend their remaining time without regret. As future medical professionals, it is crucial for medical students to establish a positive attitude toward hospice care and a correct attitude toward death. This study found that the end-of-life care attitude of medical students was more positive (103.98 ± 9.64) than that of medical students surveyed in Anhui Health Vocational and Technical College in 2018 (101.26 ± 9.74) [17] and medical students surveyed in a medical college in Zibo City in 2022 (82.49 ± 16.50) [18]. This indicates that Chinese medical students are becoming increasingly positive toward hospice care, and the development of hospice care education is steadily advancing. Medical students tended to accept death neutrally, which was consistent with the research results of Shi Wenwen [19] and Huiwu Han [20] et al. Certain research indicates that medical students in Iran generally accept death as a natural occurrence [21].

This study revealed that only children had a more positive attitude toward hospice care, while medical students who were willing to take care of dying patients had a more positive attitude toward hospice care. The tendency of death attitude has the greatest influence on hospice care attitude, and all dimensions of death attitude will affect the hospice care attitude. Other research indicates that interaction with dying patients [22], courses, and practical training on dying and death can enhance the enthusiasm for hospice care attitudes [23]. Gender, age, and professional background (clinical, nursing, etc.) are known to influence the attitude toward hospice care [24], while psychological characteristics [25] and religious beliefs [26] may also play significant roles.

The multifaceted nature of death attitude renders its affecting components more intricate: (1) Males are more fearful of death than females and tend to escape and avoid, while females are more inclined to accept death than males. In previous studies, the influence of gender differences has not been conclusively determined in different cultures and research samples [24]. (2) Individuals raised in urban environments are more prone to accept death than those raised in rural environments. This may be related to the different environments between urban and rural areas in China, where individuals in rural areas possess an insufficient understanding of death. (3) The only child is more fearful of death than the non-only child, and tends to accept death, potentially influenced by familial dynamics. Some studies have shown that the family environment plays a subtle role in shaping the attitude toward death. The open discussion of death among family members is an important factor affecting medical students’ attitude toward death [24]. (4) Those with lower education attainment are more predisposed to accept death. This finding is consistent with previous studies [24], indicating that this relationship is not simply linear and the results are conflicting, which may be related to the academic pressure faced by different grades, the degree of clinical exposure, and the curriculum. (5) It is quite abnormal that those who have engaged in hospice care- and death-related courses are more inclined to avoid death and accept death, which may be attributable to the pedagogical content and methods employed in educational settings, failing to cultivate an accurate perspective on death among students. Previous studies have shown that structured death education, palliative care, and hospice care curricula can significantly improve medical students’ attitudes toward death, such as reducing death anxiety and promoting neutral acceptance and approach acceptance of death. These curricula can help students deal with the emotional impact of death and build professional coping mechanisms [24]. Consequently, when imparting death education and related subjects in educational institutions, it is imperative to consider students’ comprehension and pertinence of teaching methods to prevent contradictory phenomena. (6) Whether or not a person has experienced the death of a relative or friend, and the emotional experience that these experiences bring, profoundly affect their perception of death [24]. Those who have taken care of terminal patients and their families have a lower tendency to neutral acceptance and a higher tendency to approach acceptance. In a similar vein, an increased number of relatives lost in the preceding year correlates with more difficulty in naturally accepting death, as well as a heightened inclination toward both acceptance and avoidance of death. This suggests that for medical students, encountering mortality is not uncommon. This indicates that while medical students may experience a shift in their perspective on mortality, it is essential to provide them with an accurate understanding of death. Medical students require time to assimilate and contemplate the information. New experiences, such as the death of a loved one and caring for end-stage patients and their families, will affect their psychological states and cognition because this study indicates that the five dimensions of death attitude are affected by current experience. The scores in the four dimensions of fear of death, death avoidance, approach acceptance, and escape acceptance ranged from high to low: I currently have a terminally ill loved one (life expectancy is less than 6 months) > I just experienced the death of a loved one > I currently have no terminally ill loved one. Conversely, regarding the neutral acceptance dimension, attitudes toward death and end of life may improve following a pertinent experience. Further research is needed to determine whether the experience of a loved one’s death and the experience of caring for a terminally ill loved one will affect medical students’ attitudes toward death, and to explore the extent of this influence and the intervention methods.

This study presents a substantial correlation between death attitude and hospice care attitude. The five dimensions of death attitude, including death fear, death avoidance, neutral acceptance, approach acceptance, and escape acceptance, are significantly positively correlated with hospice care attitude, which is different from the findings reported in 2016 [14]. The different research results may be attributed to the different survey objects. Our study examined the population of medical students nationwide, highlighting significant variability. This disparity may also stem from an increase in medical knowledge among students, resulting in a dissonance between their attitudes toward death and hospice care, of which they remain largely unaware.

## 5. Suggestions

### 5.1. Death Education and Hospice Care Courses Should Be Strengthened

It is essential to integrate ethics, psychology, sociology, and other interdisciplinary content, strengthen students’ ability to manage death and hospice care, improve teacher training and support [27], add relevant elective courses, expand new programs [28], and provide practical clinical teaching and simulation training, thereby enabling students to engage with hospice care scenarios in a secure environment, supplemented by reflective learning. Simultaneously, continuous improvement and feedback facilitate the optimization of instructional content and methodologies to guarantee superior and pragmatic education. Mandatory Modules on End-of-Life Communication. These courses must cover core competencies such as breaking bad news, discussing prognoses with honesty and empathy, navigating difficult family dynamics, eliciting patient values and preferences for care, and managing emotional responses from both patients and oneself [29]. The development of culturally specific communication training programs is particularly vital to address unique Chinese contexts.

### 5.2. Consider the Individual Differences and Psychological Support of Medical Students

Based on individual differences such as gender, profession, and experience level, such as the escape and fear of death for males, to carry out education on life and death and hospice care education, specific interventions need to be designed [4]. Concurrently, counseling should be offered to medical students to assist them in confronting death. Supporting relatives caring for dying patients, this approach aims to equip medical students to manage death anxiety, fear, and sorrow [25]. Ultimately, such methods will foster a proper understanding of death and a constructive perspective on hospice care.

### 5.3. Encourage Experience Sharing

Conduct experience-sharing sessions on death and hospice care, inviting seasoned educators or students to discuss their methods for confronting fear and avoidance, ultimately leading to acceptance. From the vantage point of an educator or a medical student, we can examine the ethical dilemmas associated with dying and hospice care in a more pragmatic manner, thereby enhancing students’ capacity for empathy and alleviating their adverse emotions. Students may also be encouraged to engage in hospice volunteer activities pertinent to their experience.

### 5.4. Establish Professional Training for Hospice Care

Currently, there is a scarcity of pertinent hospice care training in China. We can refer to the ELNEC and EPEC programs of the United States [30], which design systematic courses for nurses and doctors, respectively, whereas the PEACE plan of Japan emphasizes symptom management and communication [31]. Hospice care training mainly includes symptom management (pain control and other common symptoms) [32], communication skills (emphasis on empathy and active listening) [33], ethical and legal considerations [32], psychological and spiritual support [32], and cultural sensitivity [32]. The training modalities are highly diverse, including seminars, case analyses, video learning, role-playing, simulation training, and more [33]. The training effect is significant, enhancing the knowledge, communication skills, empathy, and self-confidence of the training staff [34]. The other, equally critical goal is to provide ongoing support for these individuals once they become practicing health professionals. The psychological toll of consistently working with suffering and death is immense. However, there are no widespread implementations of robust, structured support programs within Chinese hospitals. There is a notable lack of detailed reports on the specific content and evaluation of psychological support schemes in hospitals. To implement hospice care training for medical students, we can simplify and localize it to make it more suitable for the cultural background of mainland China. We also need to avoid the problems of insufficient class hours, the disconnect between theory and practice, and resistance from the organizational culture [35]. This also requires further research on how to improve the hospice care attitude and death attitude of medical students in the Chinese mainland and how to improve the effectiveness of relevant training and courses in the future.

## 6. Conclusions

The examination of medical students’ attitudes about hospice care and death in China reveals that the advancement of hospice care education in the country remains promising. The attitude of medical students toward hospice care has improved compared to the past. Nonetheless, this study also identified numerous deficiencies requiring correction. Specifically, the instruction of hospice care and death education necessitates prompt feedback and refinement to prevent inefficiency. Attention must be paid to the individual characteristics and psychological well-being of prospective medical personnel, making accurate education and advice imperative. By fostering healthy, positive attitudes among medical students toward hospice care and death, the development of hospice care can mature sufficiently to address the challenges posed by an aging population, thereby enhancing the future quality of medical services.

## Figures and Tables

**Table 1:** General information on medical students.

Variable	N (%)
Sex	
Male	192 (33.8%)
Female	376 (66.2%)
Birthplace	
Urban	262 (46.1%)
Rural	306 (53.9%)
Only child or not	
Yes	202 (35.6%)
No	366 (64.4%)
Highest degree held	
Junior college	12 (2.1%)
Undergraduate	528 (92.0%)
Graduate student	28 (4.9%)
Willingness to study medicine	
Yes	457 (80.5%)
No	111 (19.5%)
Willingness to care for terminally ill patients	
Yes	422 (74.3%)
No	146 (25.7%)
Previous education on death and dying	
Taken specialized courses	101 (17.8%)
Taken related topics in other courses	326 (57.4%)
Never taken related courses	141 (24.8%)
Previous experience in end-of-life care	
Yes	115 (20.2%)
No	453 (79.8%)
Previous experience with loss within one year	
Lost a relative	136 (23.9%)
Lost many relatives	49 (8.6%)
No previous experience	383 (67.4%)
Present experience	
Have lost a relative recently	49 (8.6%)
Have a terminally ill relative (life expectancy 1 year or less)	39 (8.6%)
Have no terminally ill relative	480 (84.5%)

**Table 2:** Medical students’ attitude toward death.

	Fear of Death	Death Avoidance	Neutral Acceptance	Approach Acceptance	Escape Acceptance
**Average**	2.89	2.90	4.04	2.82	2.90
**SD**	0.82	0.87	0.57	0.74	0.89

**Table 3:** The influence of medical students’ general information on the attitude toward death and hospice care.

Variables	FATCOD-B Score	Fear of Death	Death Avoidance	Neutral Acceptance	Approach Acceptance	Escape Acceptance
Sex						
Male	103.83 ± 10.91	2.98 ± 0.83	3.09 ± 0.89	3.94 ± 0.65	2.86 ± 0.79	2.89 ± 0.96
Female	104.06 ± 8.95	2.81 ± 0.71	2.80 ± 0.84	4.08 ± 0.52	2.80 ± 0.70	2.90 ± 0.86
* p*	0.801	0.015 *	<0.001 *	0.010 *	0.382	0.880
Birthplace						
Urban	103.61 ± 9.51	2.84 ± 0.73	2.92 ± 0.81	3.99 ± 0.56	2.83 ± 0.68	2.89 ± 0.84
Rural	104.41 ± 9.80	2.91 ± 0.78	2.87 ± 0.93	4.09 ± 0.57	2.81 ± 0.79	2.90 ± 0.95
* p*	0.324	0.217	0.542	0.038 *	0.708	0.849
Only child or not						
Yes	105.18 ± 10.14	2.97 ± 0.80	2.89 ± 0.94	4.09 ± 0.58	2.88 ± 0.86	3.00 ± 0.98
No	103.32 ± 9.31	2.82 ± 0.72	2.90 ± 0.83	4.01 ± 0.56	2.79 ± 0.65	2.84 ± 0.83
* p*	0.028 *	0.019 *	0.805	0.105	0.172	0.051 *
Highest degree held						
Junior college	101.58 ± 10.08	2.81 ± 0.81	3.15 ± 1.10	3.88 ± 0.58	3.23 ± 0.76	3.26 ± 0.97
Undergraduate	104.12 ± 9.57	2.88 ± 0.75	2.90 ± 0.87	4.05 ± 0.56	2.82 ± 0.74	2.90 ± 0.90
Graduate student	102.36 ± 10.92	2.69 ± 0.66	2.88 ± 0.81	3.93 ± 0.70	2.60 ± 0.50	2.81 ± 0.76
* p*	0.440	0.389	0.605	0.357	0.076 *	0.311
Willingness to study medicine						
Yes	104.16 ± 9.47	2.88 ± 0.76	2.92 ± 0.89	4.06 ± 0.55	2.82 ± 0.74	2.89 ± 0.90
No	103.23 ± 10.33	2.85 ± 0.74	2.82 ± 0.76	3.94 ± 0.65	2.80 ± 0.70	2.91 ± 0.87
* p*	0.364	0.781	0.275	0.057 *	0.767	0.853
Willingness to care for terminally ill patients						
Yes	106.29 ± 9.09	2.86 ± 0.76	2.90 ± 0.87	4.05 ± 0.57	2.85 ± 0.75	2.88 ± 0.90
No	103.18 ± 9.71	2.90 ± 0.73	2.90 ± 0.87	4.00 ± 0.56	2.73 ± 0.70	2.95 ± 0.87
* p*	<0.001 *	0.548	0.956	0.316	0.096 *	0.430
Previous education on death and dying						
Taken specialized courses	105.06 ± 13.87	3.05 ± 0.90	3.16 ± 1.00	4.02 ± 0.56	3.09 ± 0.82	2.99 ± 0.96
Taken related topics in other courses	103.91 ± 8.24	2.82 ± 0.71	2.79 ± 0.83	4.07 ± 0.55	2.80 ± 0.69	2.92 ± 0.88
Never taken related courses	103.36 ± 8.98	2.86 ± 0.73	2.96 ± 0.81	3.97 ± 0.61	2.67 ± 0.72	2.77 ± 0.86
* p*	0.935	0.033 *	<0.001 *	0.227	<0.001 *	0.131
Previous experience in end-of-life care						
Yes	102.76 ± 10.74	2.99 ± 0.78	3.03 ± 0.95	3.89 ± 0.58	2.95 ± 0.79	2.98 ± 0.92
No	104.29 ± 9.34	2.84 ± 0.74	2.87 ± 0.84	4.07 ± 0.56	2.79 ± 0.72	2.87 ± 0.88
* p*	0.128	0.056 *	0.075 *	0.002 *	0.040 *	0.274
Previous experience with loss within one year						
Lost a relative	104.22 ± 8.78	2.88 ± 0.69	2.98 ± 0.84	3.96 ± 0.56	2.81 ± 0.71	2.86 ± 0.84
Lost many relatives	100.96 ± 11.83	3.00 ± 0.90	3.01 ± 0.88	3.86 ± 0.62	3.07 ± 0.78	3.33 ± 0.97
No previous experience	104.28 ± 9.59	2.85 ± 0.75	2.85 ± 0.88	4.08 ± 0.56	2.79 ± 0.73	2.85 ± 0.89
* p*	0.072 *	0.419	0.245	0.008 *	0.037 *	0.002 *
Present experience						
Have lost a relative recently	103.49 ± 11.03	3.05 ± 0.65	2.98 ± 0.77	3.92 ± 0.50	2.87 ± 0.75	3.09 ± 0.87
Have a terminally ill relative (life expectancy 1 year or less)	103.85 ± 11.49	3.23 ± 0.84	3.20 ± 0.76	3.80 ± 0.70	3.25 ± 0.71	3.33 ± 0.83
Have no terminally ill relative	104.04 ± 9.35	2.82 ± 0.75	2.86 ± 0.88	4.06 ± 0.56	2.78 ± 0.72	2.84 ± 0.89
* p*	0.926	0.001 *	0.050	0.007 *	<0.001 *	0.001 *

*. At the 0.1 level, the correlation is significant.

**Table 4:** Correlation analysis between medical students’ attitude toward death and attitude toward hospice care.

Variables	FATCOD-B Score
Fear of death	
Pearson correlation	0.375 **
Sig (two-tailed)	0.000
Death avoidance	
Pearson correlation	0.337 **
Sig (two-tailed)	0.000
Neutral acceptance	
Pearson correlation	0.246 **
Sig (two-tailed)	0.000
Approach acceptance	
Pearson correlation	0.232 **
Sig (two-tailed)	0.000
Escape acceptance	
Pearson correlation	0.186 **
Sig (two-tailed)	0.000

**. At the 0.01 level (two-tailed), the correlation is significant.

**Table 5:** First multiple linear regression analysis of influencing factors in medical students’ attitude toward hospice care.

Variables	B	Std. Error	Standardized Coefficient (Beta)	t	*p*
Fear of death	4.272	0.699	0.334	6.114	0.001
Death avoidance	1.850	0.564	0.167	3.281	0.001
Neutral acceptance	6.312	0.626	0.372	10.091	0.001
Willingness to care for the dying	3.328	0.783	0.151	4.253	0.001
Only child or not	−0.800	0.720	−0.04	−1.111	0.267
Approach acceptance	0.311	0.620	0.024	0.502	0.616
Escape acceptance	0.267	0.481	0.025	0.556	0.578

**Table 6:** Second multiple linear regression analysis of influencing factors in medical students’ attitude toward hospice care.

Variables	B	Std. Error	Standardized Coefficient (Beta)	t	*p*
Fear of death	4.690	0.629	0.367	7.453	0.001
Death avoidance	1.751	0.535	0.158	3.273	0.001
Neutral acceptance	6.448	0.618	0.380	10.431	0.001
Willingness to care for the dying	3.256	0.775	0.148	4.200	0.001

## Data Availability

The raw data supporting the conclusions of this article will be made available by the authors on request.
